# Structural insights into the function, dysfunction and modulation of Kv3 channels

**DOI:** 10.1016/j.neuropharm.2025.110483

**Published:** 2025-04-25

**Authors:** Manuel Covarrubias, Qiansheng Liang, Linh Nguyen-Phuong, Kyle J. Kennedy, Tyler D. Alexander, Andrew Sam

**Affiliations:** a Department of Neuroscience, Sidney Kimmel Medical College of Thomas Jefferson University, Bluemle Life Science Building, 233 South 10th Street, Room 231, Philadelphia, PA, 19107, USA; b Vickie and Jack Farber Institute for Neuroscience, USA; c Jefferson Synaptic Biology Center, USA

**Keywords:** Kv channel, Cryo-EM, Kv channel gating, Kv channel modulators, Pain modulation

## Abstract

The third subfamily of voltage-gated K^+^ (Kv) channels includes four members, Kv3.1, Kv3.2, Kv3.3 and Kv3.4. Fast gating and activation at relatively depolarized membrane potentials allows Kv3 channels to be major drivers of fast action potential repolarization in the nervous system. Consequently, they help determine the fast-spiking phenotype of inhibitory interneurons and regulate fast synaptic transmission at glutamatergic synapses and the neuromuscular junction. Recent studies from our group and a team of collaborators have used cryo-EM to demonstrate the surprising gating role of the Kv3.1 cytoplasmic T1 domain, the structural basis of a developmental epileptic encephalopathy caused by the Kv3.2-C125Y variant and the mechanism of action of positive allosteric modulators involving unexpected interactions and conformational changes in Kv3.1 and Kv3.2. Furthermore, our recent work has shown that Kv3.4 regulates use-dependent spike broadening in a manner that depends on gating modulation by phosphorylation of the channel’s N-terminal inactivation domain, which can impact activity-dependent synaptic facilitation. Here, we review and integrate these studies to provide a perspective on our current understanding of Kv3 channel function, dysfunction and pain modulation in the nervous system.

## 1. Introduction

Voltage-gated K (Kv) channels play essential roles in the nervous system of diverse organisms by shaping action potential (AP) waveforms and regulating spike latency and patterns of repetitive spiking (Jan and Jan, 2012, Johnston et al., 2010). The diverse voltage- and time-dependent properties of Kv channels help generate a broad spectrum of spiking properties necessary to encode meaningful electrical signaling in the nervous system. In mammalian organisms, there are twelve gene subfamilies encoding a total of forty Kv channel subunits, each including a voltage sensing domain (VSD) and a pore domain (PD) (Gutman et al., 2003; Kim and Nimigean, 2016). All Kv channels are tetrameric assemblies made of homologous subunits, which generate two distinct configurations (Kim and Nimigean, 2016). Whereas Kv1, Kv2, Kv3, Kv4 and Kv7 channels exhibit domain swapped architectures (Chi et al., 2022, Fernandez-Marino et al., 2023, Kise et al., 2021, Sun and MacKinnon, 2020, Tan et al., 2022, Ye et al., 2022), Kv10, Kv11, and Kv12 channels lack this feature (Barros et al., 2020). Kv5, Kv6, Kv8, and Kv9 subunits are also especial because they are electrically silent (i.e., do not form ion conducting channels) but are compatible with Kv2 subunits (Bocksteins and Snyders, 2012). Thus, non-conducting Kv channel subunits co-assemble selectively with Kv2 channel subunits to form heterotetramers with voltage-dependent properties that are distinct from those of Kv2 homotetramers (Bocksteins, 2016).

Kv3 channels in mammals are closely related to the *Shaw* gene from *Drosophila melanogaster*, which was the first Kv3 gene to be discovered and cloned (Wei et al., 1990). Shortly thereafter, the mammalian Kv3 orthologs were also cloned, heterologously expressed and characterized (McCormack et al., 1990; Rudy et al., 1991). The Kv3 subfamily includes four genes, *KCNC1, KCNC2, KCNC3 and KCNC4*, which encode Kv3.1, Kv3.2, Kv3.3 and Kv3.4, respectively (Gutman et al., 2003; Kaczmarek and Zhang, 2017). Kv3 channels are expressed discretely in central and peripheral neurons and are generally localized in somata, axons and synaptic boutons (Chang et al., 2007; Kaczmarek and Zhang, 2017; Rudy et al., 1992; Rudy and McBain, 2001; Trimmer, 2015; Weiser et al. 1994, 1995). Kv3.3 channels are also found in these compartments and, additionally, in dendrites (Choudhury et al., 2020; Kaczmarek and Zhang, 2017; Richardson et al., 2022; Zagha et al., 2010; Zhang and Kaczmarek, 2016). The number of possible Kv3 isoforms is increased further by gene splicing, which mainly affects the variable C-terminal region of the protein (Kaczmarek and Zhang, 2017; Ozaita et al., 2002; Ponce et al., 1997; Rudy et al., 1999; Rudy and McBain, 2001). This posttranscriptional processing expands the diversity of possible heterotetramers exhibiting differential distribution, biophysical properties and modulation.

Kv3 channels are high-voltage activating and exhibit fast kinetics of activation and deactivation (Joho and Hurlock, 2009; Rudy and McBain, 2001; Wu et al., 2021). Therefore, they are well suited to repolarize APs, a function that depends on their ability to generate a timely resurgent current as the membrane potential repolarizes (Labro et al., 2015). In fast spiking interneurons, Kv3 channels contribute to a brief but significant afterhyperpolarization (AHP) that plays two major roles (Kaczmarek and Zhang, 2017; Rudy and McBain, 2001). First, it promotes recovery from inactivation of voltage-gated Na (Nav) channels to restore the fraction of Nav channels that is necessary to initiate another AP shortly after the previous AP. Second, to facilitate AP initiation and maintain a short inter-spike interval, fast Kv3 channel deactivation ensures the short duration of the AHP, which would otherwise oppose the necessary depolarization to reach AP threshold. Recent studies have demonstrated that acquired and congenital Kv3 dysfunction can lead to significant, neurological, neurodevelopmental and behavioral abnormalities (Anderson et al., 2018; Cameron et al., 2019; Chien et al., 2007; Clatot et al. 2023, 2024; Gunthorpe, 2022; Joho and Hurlock, 2009; Mukherjee et al., 2022; Park et al., 2019; Rademacher et al., 2020; Ritter et al. 2015a, 2015b; Schwarz et al., 2022; Yanagi et al., 2014; Yeap et al., 2022; Zemel et al. 2017, 2018; Zhang and Kaczmarek, 2016). These developments have sparked a growing interest in elucidating the structure, function and modulation of Kv3 channels and a search for novel therapeutic targets.

Excellent review articles published previously have broadly discussed Kv3 channels (Joho and Hurlock, 2009; Kaczmarek and Zhang, 2017; Rudy et al., 1999; Rudy and McBain, 2001; Zemel et al., 2018; Zhang and Kaczmarek, 2016). This review article focuses on major advances emerging from our recent work, which concerns the following topics: 1) the cryo-EM structures of hKv3.1a in the apo conformation and conformations adopted upon binding of two positive allosteric modulators; 2) the structural basis of a Kv3.2 encephalopathy; and 3) how the plasticity of use- and frequency-dependent AP broadening is dependent on modulation of Kv3.4 fast inactivation by phosphorylation of its N-terminal inactivation domain (NTID) (Alexander et al. 2022, 2025; Chi et al., 2022; Clatot et al., 2024; Feng et al., 2024; Liang et al., 2024).

## 2. The cryo-EM structure of hKv3.1a revealed gating control by the cytoplasmic T1 domain

According to the canonical electromechanical model of Kv channel gating, the S4 transmembrane segment of the VSD from each subunit of the channel’s tetrameric assembly undergoes an outward helical screw movement in response to a depolarization of the membrane potential. This is analogous to the *sliding helix* model of voltage-sensing originally proposed by Bill Catterall, 1986, 2010. As a result, 3–4 positive charges carried by the side chains of arginine residues located at every third position within the transmembrane S4 helix are translocated outward across the focused membrane electric field at the location of a phenylalanine in the transmembrane S2 helix, which constitutes the hydrophobic plug of the gating pore (i.e., the charge transfer center, CTC). Once all four S4 helices have adopted the activated conformation, displacements of the intracellular S4-S5 linkers trigger a concerted conformational change at the bundle crossing of the transmembrane S6 helices in the PD, opening the intracellular gate and allowing K^+^ conduction (Cowgill and Chanda, 2021; Kim and Nimigean, 2016). In non-canonical models, the transmembrane S5 helix may also contribute to propagated rearrangements that help expand of the S6 bundle crossing to open the gate (Fernandez-Marino et al., 2018). Although these models generally account for the way in which voltage-gated ion channels use the electrical energy stored in the membrane potential to control their open probability, they do not account for the mechanisms that tailor the specific functions of diverse Kv channels and how their specific gating properties are controlled. For instance, Kv3 channels exhibit ultrafast voltage-dependent activation and deactivation, making them optimally suited to quickly repolarize the AP and allow repetitive spiking at rates of the order of hundreds of Hz. Several early studies and the recent publication of the cryo-EM structure of hKv3.1a at a resolution of 3.1 Å shed light on the structural basis of gating control by the cytoplasmic N-terminal region of Kv channels (Chi et al., 2022; Cushman et al., 2000; Minor et al., 2000; Wang and Covarrubias, 2006; Wang et al., 2005).

The cT1D constitutes a major part of the N-terminal region of Kv1, Kv2, Kv3 and Kv4 channels, which is involved in multiple interactions with structural and functional significance (Chi et al., 2022; Fernandez-Marino et al., 2023; Kise et al., 2021; Tan et al., 2022; Ye et al., 2022). For instance, it determines the subfamily specific tetramerization among members of the same Kv channel subfamily (Bixby et al., 1999; Li et al., 1992). Additionally, it provides docking surfaces for accessory subunits and interactions with the cytoplasmic C-terminal region to regulate axonal trafficking (Gulbis et al., 2000; Kaczmarek and Zhang, 2017; Kise et al., 2021; Long et al., 2005; Pioletti et al., 2006; Xu et al., 2007). Furthermore, multiple lines of evidence have strongly suggested direct gating roles for discrete cT1D regions near the intracellular gating machinery of Kv1 and Kv4 channels (Cushman et al., 2000; Kise et al., 2021; Minor et al., 2000; Strang et al., 2001; Wang and Covarrubias, 2006; Wang et al., 2005). One particular study demonstrated voltage-dependent changes in the accessibility of thiol specific reagents to modify native cysteines in the cT1D Zn^++^ site, which precisely follow the voltage-dependent conductance change of Kv4.1 channels (Wang and Covarrubias, 2006). It is also significant that the cT1Ds of Kv2, Kv3 and Kv4 channels have a conserved intersubunit Zn^++^ binding site, which helps stabilize the cT1D assembly and may serve as a redox sensor that modulates gating (Bixby et al., 1999; Wang et al., 2007a). To help establish a direct contribution of the cT1D to voltage-dependent gating, however, it was necessary to visualize the underlying interactions with near-atomic resolution.

**Fig. 1.**
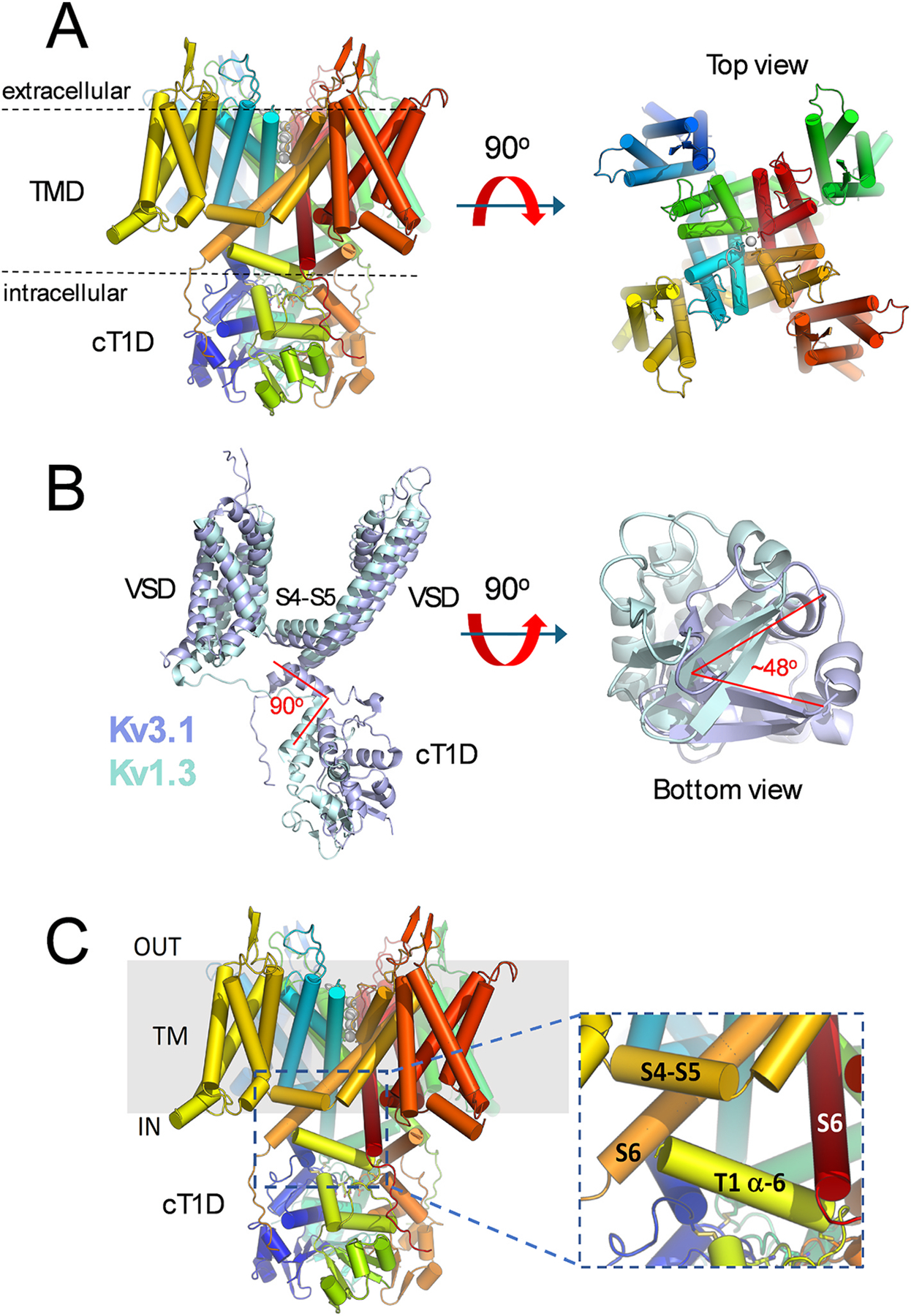
Cryo-EM apo structure of hKv3.1a in an open conformation. **A**) Lateral and outside views of the hKv3.1a tetrameric assembly (PDB ID: 7PHI) depicting the transmembrane domain (TMD) encompassing the VSD and PD, and the cytoplasmic T1 domain (cT1D). The top view demonstrates the domain-swapped architecture of the tetramer, a feature that is characteristic of Kv1, Kv2, Kv3, Kv4 and Kv7 channels. **B**) Single subunit overlay of the hKv3.1a and Kv1.3 structures. While there is an excellent agreement between the transmembrane domains of the two structures, the conformations of their cT1Ds differ substantially. Relative to each other, a bottom view reveals a 48° rotation. Also, the α–6 helix of Kv3.1 is rotated 90° inward, relative to the position of the equivalent segment in Kv1.3. **C)** Zoomed in view of the cT1D α–6 helix, which together with the S4-S5 linker and the distal segment of the S6 helix forms the gating triad that controls fast voltage-dependent gating. These visualizations were created in PyMOL (The PyMOL Molecular Graphics System, Version 3.0 Schrödinger, LLC).

The cryo-EM structure of hKv3.1a at a resolution of 3.1 Å revealed a four-fold symmetric assembly, with general features like those of Kv1 channels in a putative open conformation (Fig. 1) (Chi et al., 2022). The hKv3.1a oligomers display a cT1D assembly, with the membrane-spanning VSD from each subunit connected to the corresponding cT1D via the T1-S1 linker. The VSD encompasses helices S1-S4, which are connected to a PD by the S4-S5 linker on the intracellular face of the oligomer (Fig. 1). The PD includes the S5 helix, the selectivity filter and pore helix, and the S6 helix. The protein backbone in the selectivity filter and the S6 helices line the narrow pore and the central cavity of the permeation pathway, respectively. As a result of the domain-swapped architecture of the overall structure, the VSD from a given subunit interacts with the PD of the neighboring subunit, creating an assembly with a central pore surrounded by four VSDs. Consistent with an activated open conformation, three arginine side chains from the S4 helix are positioned outwardly with respect to the CTC and the S6 bundle crossing is wide open.

Major differences between the structure of hKv3.1a and those of Kv1-type channels emerged, however, from a closer examination of the relationship between the cT1D and the transmembrane domains (VSD and PD) (Chi et al., 2022). The cT1D of hKv3.1a is rotated about 48° clockwise in relation to the cT1D of the Kv1.2 and Kv1.3 channels (Fig. 1). Furthermore, the α–6 helix of the cT1D is rotated 90° inward with respect to the same segment in Kv1.2 and Kv1.3, bringing the α–6 helix closer to the S4-S5 linker and the distal segment of the S6 helix (S6 tail, S6T) (Fig. 1). Therefore, four interacting subunits of the hKv3.1a assembly bring together the α–6 helix, the S4-S5 linker and the S6T to form one gating triad per subunit, where a network of interactions have been optimized to control electromechanical transduction and pore opening (Chi et al., 2022). Interactions in the gating triad include two acidic residues (E116 and D120) at the C-terminal end of the α–6 helix, which form salt bridges with K449 in S6T. At the other end of the α–6 helix, W106 and M107 establish non-polar interactions with A448 of the S6T. Also, because the C-terminal end of the α–6 helix is rich in acidic residues, it may contribute to coulombic interactions with an excess of S4-S5 linker positive charges carried by R332, H336 and R339. To validate these interactions, systematic mutational perturbations of key residues of the gating triad produced major changes in the observed rate constants of voltage-dependent activation and deactivation (Chi et al., 2022). The Kv3.1 gating triad described here is distinct from the previously discovered interfacial gating triad in a Shaker Kv channel (Chowdhury et al., 2014). Chowdhury et al. showed that electromechanical coupling is dictated by interactions between three critical amino acid side chains (R394 and E395 in the S4-S5 linker and a Y485 in the S6T). By contrast, the gating triad involving the cT1D is formed by a network of interactions between three distinct segments, the S4-S5 linker, the S6T and the α–6 helix of the T1 domain. It is also significant that the α–6 helix is in the proximity of the intersubunit Zn^++^ binding site, which may undergo rearrangements coupled to voltage-dependent gating in the PD. Separately, an acidic residue in the T1 domain (D81) may help determine the relatively large unitary conductance of Kv3.1 and create a region with a negative electrostatic potential that could prevent local K^+^ depletion over periods of fast repetitive spiking (Chi et al., 2022).

## 3. The atomic structural basis of a developmental epileptic encephalopathy

A growing number of Kv3.1, Kv3.2, and Kv3.3 variants have been linked to a spectrum of severe encephalopathies and psychiatric disorders, and mapped on the structure of hKv3.1a and models of Kv3.2 (Chi et al., 2022; Faulkner et al., 2024; Gunthorpe, 2022; Schwarz et al., 2022). The patients affected by these variants are mostly children and young adults, who exhibit ataxia, seizures, autistic behaviors, intellectual disability and neurodevelopmental delay (Cameron et al., 2019; Clatot et al., 2023; Muona et al., 2015; Oliver et al., 2017; Park et al., 2019; Zhang and Kaczmarek, 2016). Although most Kv3.1 and Kv3.2 variants are de novo, the voltage sensor variant Kv3.1-R320H is recurrent and is responsible for progressive myoclonus epilepsy type 7 (EPM7; PME7) (Muona et al., 2015; Oliver et al., 2017). Kv3.3 pathogenic variants, which cause spinocerebellar ataxia 13 (SCA13), have similar voltage sensor mutations (R420H and R423H) and other regions, including the N-terminus, the S1, S5 and S6 segments, and the C-terminus (Zhang and Kaczmarek, 2016). So far, no pathogenic Kv3.4 variants have been found. However, a pathogenic variant of MiRP2 (*KCNE3*), a promiscuous accessory subunit that modulates Kv3.4 channels through a direct interaction, has been linked to a case of periodic paralysis (Abbott et al. 2001, 2006).

Pathogenic variants of Kv3.1 and Kv3.2 have point mutations mostly located within membrane spanning helices and linkers canonically involved in voltage-dependent gating and ion permeation, such as the VSD and the PD (Faulkner et al., 2024; Gunthorpe, 2022). Many of these mutations are especially clustered within the transmembrane S6 helix and only a handful of them have been found in the cytoplasmic C-terminal region. There is therefore a wealth of knowledge to hypothesize how the mutations located in those regions may alter gating and expression of Kv3 channels through gain-of-function (GoF) or loss-of-function (LoF) effects. In contrast, much less is known about regions that may play non-canonical gating roles and how mutations within these regions may affect function. For instance, among several recently identified Kv3.2 variants (Li et al., 2022; Mukherjee et al., 2022; Rademacher et al., 2020; Rydzanicz et al., 2021; Vetri et al., 2020; Wang et al., 2022), Kv3.2-C125Y and Kv3.2-C125W involve single substitutions in the cytoplasmic T1 domain (Clatot et al., 2024; Schwarz et al., 2022). These are GoF mutations that induce large hyperpolarizing shifts in the voltage dependence of activation, essentially transforming this Kv channel into a low-voltage activating Kv3.2 channel. Patients expressing these variants exhibit developmental epileptic encephalopathy (DEE), which is mainly characterized by neurodevelopmental abnormalities, intellectual disability and seizures (Clatot et al., 2024; Schwarz et al., 2022).

To determine the atomic structural basis of the mechanism by which these mutations induce a relative stabilization of the channel’s open state (as suggested by the hyperpolarizing shift), we leveraged the cryo-EM structure of Kv3.1a in the open conformation (Clatot et al., 2024). As reviewed above (Cryo-EM structure of hKv3.1a), this structure revealed that the α–6 helix of the cytoplasmic T1 domain together with the intracellular S4-S5 linker and the tail of the S6 helix form a specialized gating triad that controls the stability of the channel’s open state. In canonical gating models of Kv channels, membrane potential changes move the positive charges of the S4 voltage sensors across the membrane electric field. Consequently, the S4-S5 linkers are also displaced to act as molecular wrenches that control the opening and closing of the intracellular S6 bundle crossing that constitutes the activation gate. To regulate these movements in hKv3.1a, the specialized α–6 helices of the cytoplasmic T1 domain interact with both the S4-S5 linkers and the S6 tails, thereby helping to determine the fast opening (activation) and closing (deactivation) kinetics that are characteristic of Kv3 channels. For instance, speeding up closing would on average keep the activation gate closed longer, and thereby shifting the voltage dependence of activation toward more depolarized voltages. The opposite would be true if the closing of the activation gate were slowed down, resulting in a less depolarized (or even hyperpolarized) voltage dependence of activation.

**Fig. 2.**
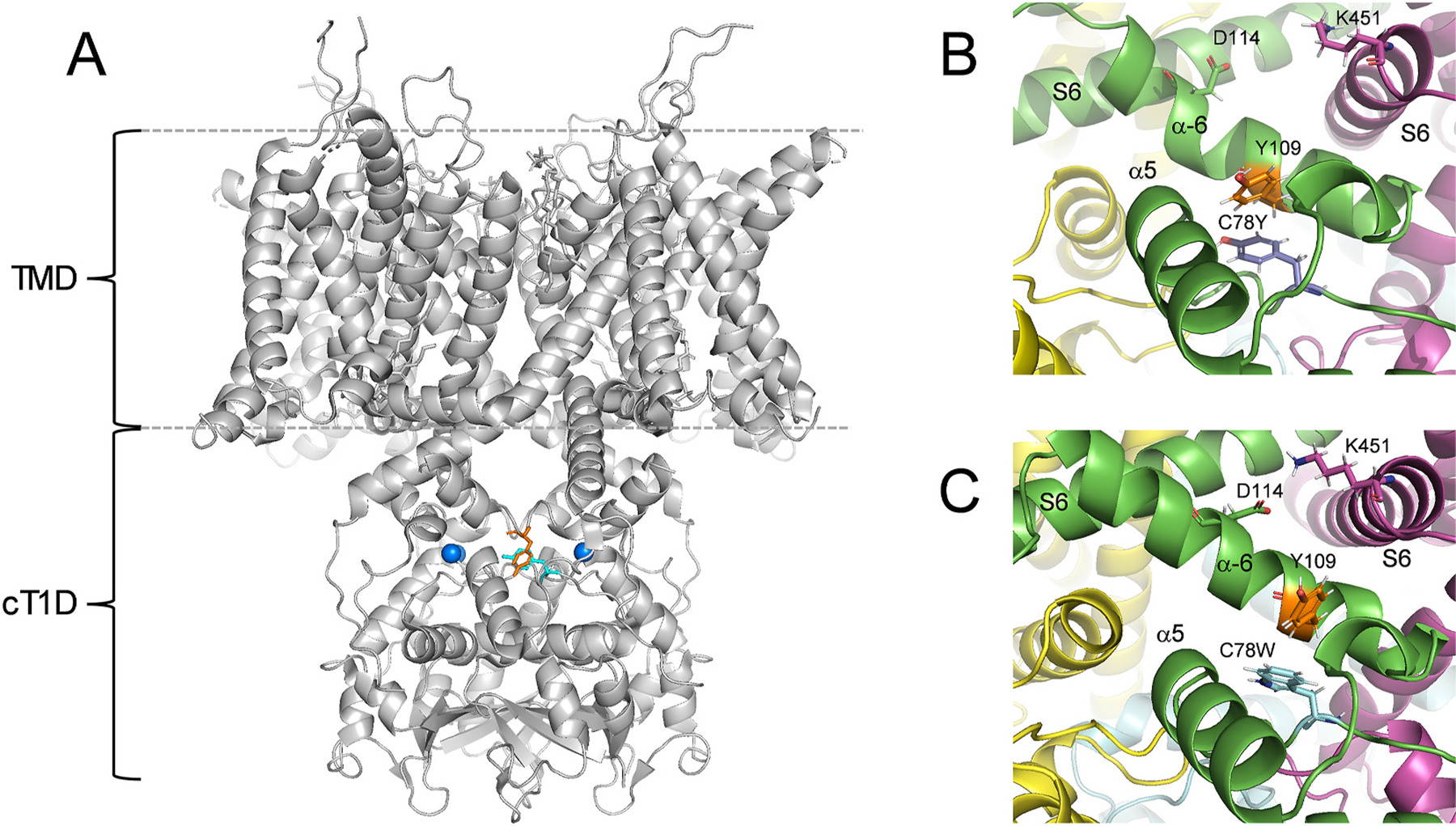
The Kv3.1-C78Y and Kv3.1-C78W exhibit a de novo Π-Π stacking interaction in the cytoplasmic T1 domain. **A)** Structural model of the Kv3.1-C78Y variant. This model was created as previously described (Clatot et al., 2024). The colored side chains of Y78 (orange) and Y109 (cyan) indicate the location of the Π-Π stacking interaction within the cT1D. As a reference, the blue spheres represent bound Zn^++^. **B)** Zoomed-in view of the Π-Π stacking interaction between Y78 and Y09 of the α–6 helix. **C)** Zoomed-in view of the Π-Π stacking interaction between W78 and Y109 of the α–6 helix. The model of Kv3.1-C78W was created using AlphaFold3 (Jumper et al., 2021).

Considering the high degree of homology between Kv3.1 and Kv3.2, it is notable that Kv3.1-C78 and Kv3.2-C125 are at the N-terminal end of the α–5 helix, directly facing Kv3.1-Y109 and Kv3.2-Y156 in the α–6 helix (from the same subunit), respectively (Fig. 2). Thus, atomistic molecular dynamics (MD) simulations of Kv3.2-C125Y revealed increased interactions between Y125 (the mutated residue) and Y156 (a native residue), which may favor a strong Π-Π stacking interaction between the phenol rings of the tyrosine side chains (Clatot et al., 2024). Increased interactions between the homologous residues (Y78 and Y109) were also observed in atomistic MD simulations of Kv3.1-C78Y, which has a biophysical phenotype like that of Kv3.2-C125Y (Fig. 2). Consequently, this interaction may impact the conformation of α–6 helix and thereby favor a crosstalk between the S4-S5 linkers and the S6 bundle crossing to stabilize the open conformation. No MD simulations with the Kv3.2-C125W variant are yet available; however, its biophysical phenotype is very similar to that of the Kv3.2-C125Y and, therefore, a similar Π-Π stacking interaction between W125 and Y156 may be primarily responsible for the GoF phenotype induced by C125W. An AlphaFold-3 model of Kv3.1-C78W is consistent with this interpretation (Fig. 2). Further MD simulation analyses rendered a change in α–6 flexibility as a defining factor of the GoF phenotype induced by Kv3.1-C78Y and Kv3.2-C125Y less likely. Nevertheless, a reorganization of a network of salt bridges involving the α–6 helix, the S4-S5 linker and the S6 tail in Kv3.1-C78Y and Kv3.2-C125Y may be secondary to the strong Π-Π stacking interaction proposed above, which is the defining factor of the GoF induced by Kv3.1-C78Y and Kv3.2-C125Y. Additional interactions and slower rearrangements may not have been captured within the relatively short timeframe (700 ns) of the MD simulations (Clatot et al., 2024).

## 4. The mechanism of action of imidazolidinedione derivatives

Kv channels have received significant attention as therapeutic targets of small molecule modulators because they are broadly implicated in disorders affecting excitable tissues, such as the brain and the heart (Busserolles et al., 2016; Castle, 2010; Faulkner et al., 2024; Gribkoff and Winquist, 2023; Gunthorpe, 2022; Johnston, 2021; Large et al., 2012; Musselman et al., 2023; Wulff et al., 2009). Generally, natural and synthetic molecules that modulate Kv channels can be grouped into two major classes (Johnston, 2021; Matsumura et al., 2021): 1) pore blockers acting at external and/or internal binding sites in the ion permeation pathway, and 2) allosteric gating modifiers acting on the voltage sensing apparatus or the pore gates. Classical Kv channel inhibitors include quaternary ammonium derivatives, such as tetraethylammonium (TEA), which act as pore blockers, and 4-aminopyridine (4-AP), which may act as pore blocker and gating modifier (Armstrong and Loboda, 2001; Johnston, 2021; Kehl, 2017). Also, researchers have discovered numerous relatively specific peptide toxins that act as either pore blockers or gating modifiers of Kv channels (Matsumura et al., 2021; Rodriguez de la Vega et al., 2003; Zhao et al., 2019). Although Kv channel modulators have been useful as tools to investigate fundamental mechanism of gating and permeation, their therapeutic potential has been hampered by a combination of factors including toxicity, limited specificity and inadequate pharmacokinetics. The specificity problem is particularly challenging because many structural and functional properties are conserved across all members of the Kv channel superfamily.

Kv3 channels are relatively hypersensitive to TEA and 4-AP (Kaczmarek and Zhang, 2017; Liu et al., 2017; Rudy and McBain, 2001; Vega-Saenz et al., 1992). Concentrations as low as 0.5–1 mM of TEA and 50–100 μM of 4-AP effectively inhibit Kv3 channels in both native and heterologous expression systems. Therefore, these compounds have been used to determine the contributions of Kv3 channels to spiking patterns and the repolarization of the AP (Bean, 2007; Muqeem et al., 2018; Rudy and McBain, 2001). However, caution is necessary when interpreting the results of these experiments because the large conductance Ca^++^-activated K^+^ channel and Kv7 channels are also blocked by low concentrations of TEA. Also, the peptides BDS-I and BDS-II, from *Anemonia sulcata* were discovered as specific inhibitors of Kv3 channels, but their specificity has been questioned because some voltage-gated Na^+^ channels are also highly sensitive to these peptides (Diochot et al., 1998; Liu et al., 2012; Martina et al., 2007; Wang et al., 2007b; Yeung et al., 2005).

While searching for specific positive modulators (openers) of Kv3 channels that may help treat hearing disorders, researchers at Autifony Therapeutics, Ltd. developed imidazolidinedione derivatives that specifically target Kv3.1 and Kv3.2 with an in vitro EC_50_ of the order of 1–3 μM (Alvaro et al., 2012, Boddum et al., 2017; Brown et al., 2016; Rosato-Siri et al., 2015). Further work has shown that these compounds may help treat a wide range of neurological and psychiatric disorders that implicate Kv3 channel dysfunction such as fragile-X syndrome, hearing disorders, schizophrenia, and progressive myoclonic epilepsy type-7 (PME-7) (Anderson et al., 2018; Angelescu et al., 2022; Brown et al., 2016; Chambers et al., 2017; El-Hassar et al., 2019; Faulkner et al., 2024; Feng et al., 2024; Glait et al., 2018; Kaar et al., 2022; Musselman et al., 2023; Parekh et al., 2018; Yanagi et al., 2014). To pave the way for the development of specific small molecule modulators of Kv3 channels with improved potency and efficacy, we determined the biophysical and structural basis of the mechanism of action and selectivity of the imidazolidinedione derivative known as AUT5 (Liang et al., 2024). This compound stabilizes the open conformation of Kv3.1 and Kv3.2 with remarkable specificity and an EC_50_ of ~3 μM (for Kv3.2) in a heterologous expression system. At 2 μM, AUT5 induces substantial positive modulation of Kv3.1 and Kv3.2. In contrast, closely related Kv channels, such as Kv1.2, Kv2.1, Kv3.4, K-Shaw, and Kv4.2 display no modulation by AUT5. The insensitivity of Kv3.4 to AUT5 modulation is especially striking because it is the closest relative of Kv3.1 and Kv3.2. This difference is significant because it provided the first clues in the search for the site of AUT5 action. A systematic analysis of the Kv3.1, Kv3.2 and Kv3.4 primary sequences and chimeras that exchanged discrete regions between Kv3.2 and Kv3.4 demonstrated that a short extracellular linker between the S5 and S6 transmembrane segments of the pore domain is the most significant determinant of the sensitivity to AUT5. Supporting the critical role of this extracellular linker, also known as the “turret”, and the molecular basis of AUT5 specificity, a sequence alignment showed that the turrets of Kv3.1 and Kv3.2 are unique and identical. The turrets of Kv3.3 and Kv3.4, by contrast, diverge significantly, and no homologous turrets are found across all members of the Kv channel superfamily (Liang et al., 2024). Despite this, the turret is not the binding site of AUT5. Detailed analyses revealed that the SAS sequence, an amino acid triad near the center of the Kv3.1 and Kv3.2 turrets critically determines the turret conformation that is necessary to confer positive modulation by AUT5. In contrast, RGN sequence is found at the equivalent positions in Kv3.4, suggesting that the Kv3.4 turret adopts a distinct conformation to resist positive modulation by AUT5.

**Fig. 3.**
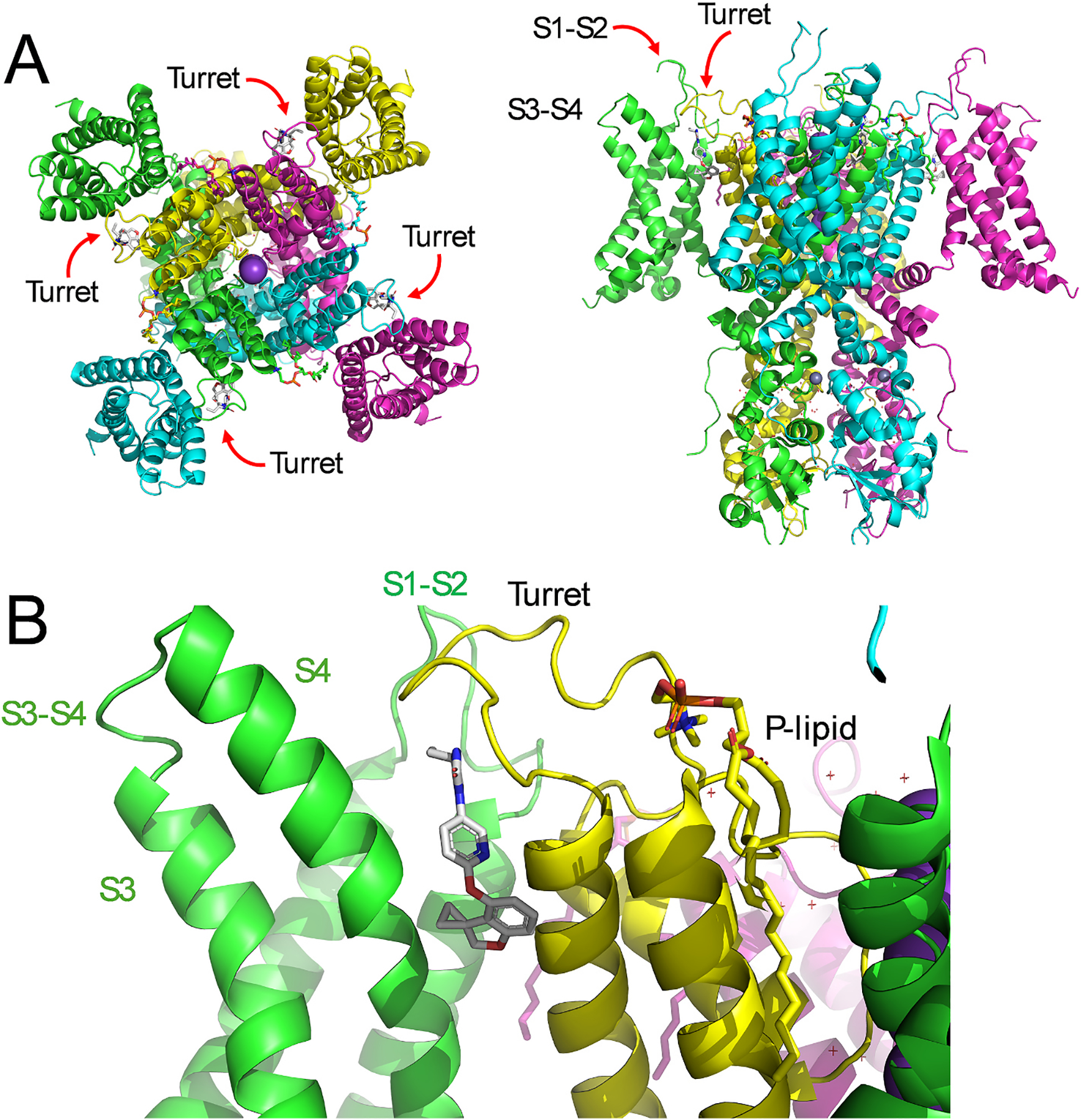
The cryo-EM structure of the AUT5-bound conformation of hKv3.1a. **A)** Outside view (left) of hKv3.1a depicting four equivalent AUT5 binding pockets. AUT5 occupies the inter-subunit interfaces between the VSD and the PD. The extracellular turrets mark the location of the binding sites. The lateral view (right) shows that AUT5 occupies a relatively deep pocket that is capped by the turret. **B)** Zoomed in view of the AUT5 binding site. AUT5 pocket is lined by the S4 helix on the VSD side and the S5 helix on the PD side. In the bound conformation, the turrets fold over the occupied site and their C-terminal sides adopt a two-turn α-helix structure. Part of the pocket is also exposed to the lipid bilayer. Using the coordinates of the AUT5 bound structure of hKv3.1a at a resolution of 2.9 Å (PDB ID: 7PHI), these visualizations were created in PyMOL (The PyMOL Molecular Graphics System, Version 3.0 Schrödinger, LLC).

The high-resolution cryo-EM structures of hKv3.1a in the apo and AUT5-bound conformations at a resolution of 2.9 Å ultimately showed the location of the binding site and revealed the mechanism of action (Fig. 3). AUT5 molecules occupy four equivalent deep pockets at the extracellular side of the tetrameric domain-swapped hKv3.1a assembly. A given binding pocket is flanked by the S4 and S5 transmembrane helices from neighboring subunits and the turret folds over the entrance to the cavity, trapping the bound AUT5 in its binding site. The presence of four equivalent binding sites is consistent with the observed apparent positive cooperativity with a Hill coefficient of ~2 (Liang et al., 2024). A closer view of the occupied pocket showed that the polar imidazoline-2, 4-dione (ID) group of AUT5 forms H-bonds with the peptide backbone atoms of R368, I369, and A370, which are on the S5 flanking region of the turret. Deeper in the pocket, the apolar spiro[2H-1-benzofuran-3, 1′-cyclopropane]-4-yl (SBC) group of AUT5 makes Van der Waals contacts with V312 and F315 in the S4 helix, and M362, Y365, and I369 in the S5 helix. This visualization is surprising and revealing because the contact residues are conserved in all Kv3 channels, suggesting that the AUT5 may bind to all of them and that the striking selective modulation of Kv3.1 and Kv3.2 is dictated by other structural factors involving differences in the turret region as noted above. A complementary cryo-EM structure showed that AUT1 (which is structurally analogous to AUT5) similarly occupies the AUT5 pocket (Liang et al., 2024). It is therefore likely that related imidazolidinedione derivatives (e.g. AUT00206) that are undergoing preclinical and clinical testing may also occupy this pocket and share the mechanism of action (Feng et al., 2024; Kaar et al., 2022; Musselman et al., 2023).

Since the hKv3.1a apo structure shows the turret in an extended unstructured conformation, we reasoned that the turret undergoes induced-fit conformational changes upon AUT5 binding to trap the bound compound (Liang et al., 2024). This conformational change is only possible in Kv3.1 and Kv3.2 because the SAS turret triad favors a structural rearrangement within the C-terminal half of the turret from unstructured to a two-turn α-helix. This change is apparently necessary for the overall change in the turret’s structure from an extended conformation to a folded conformation above the bound compound. As a result, the turret undergoes an 11 Å displacement toward the extracellular S3-S4 linker of the voltage sensor, which, in turn, is also displaced 5 Å toward the extracellular S1-S2 linker of the voltage-sensing domain. The re-organization of the interactions between the VSD and the turret is stabilized by a putative polar interaction between S1-S2 and an Asn side chain of the turret that is two residues upstream from the SAS triad. It is notable that Ser replaces Asn at this critical site in Kv3.4. We hypothesize that the ultimate consequence of these rearrangements is to immobilize the S4 voltage sensor in its UP activated conformation, which would then stabilize the open state of Kv3.1 and Kv3.2 channels and explain the positive modulation (Liang et al., 2024). Strengthening the conclusions of this work, an independent cryo-EM study reported similar binding and interactions for a distinct class of Kv3.1 positive modulators (Chen et al., 2023). This study described the binding of compound-4, a polycyclic derivative with features analogous to those of AUT1 and AUT5. Consequently, compound-4 binds like AUT1 and AUT5 to four equivalent sites facing the extracellular side of Kv3.1, making H-bond contacts with backbone groups in the external turret region and deeper contacts with non-polar amino acid side chains. Consequently, compound-4 binding also induces a turret conformational change virtually identical to that induced by AUT1 and AUT5. There is, however, an entirely distinct binding site for another class of Kv3.1 positive modulators such as Lu AG00563 (Botte et al., 2022). Facing the intracellular side of the channel, Lu AG00563 appears to occupy only one out four possible binding pockets in the Kv3.1 tetrameric assembly. In this pocket, Lu AG00563 is flanked by the side chains of residues from the S1 and S4 segments in the VSD, the S4-S5 linker and the S5 segment in the PD. Binding of Lu AG00563 in this critical location may stabilize the open conformation of the channel; however, the mechanism of action has not been elucidated.

## 5. Overview of Kv3.4 expression, structure, function, modulation and dysfunction

The Kv3.4 channel is expressed throughout the rodent central nervous system, where it may exist in heterotetramers including other Kv3 subunits (Baranauskas et al., 2003; Brooke et al. 2004a, 2004b; Joho and Hurlock, 2009; Kaczmarek and Zhang, 2017; Kim et al., 2020; Rowan and Christie, 2017; Rudy and McBain, 2001). Specific evidence for Kv3.4 expression has been found in parvalbumin interneurons, dentate granule cells of the hippocampus, globus pallidus neurons, type 2 afferent spiral ganglion neurons of the auditory system, brain stem nuclei, cerebellar stellate interneurons and motor neurons in the spinal cord. Punctate expression in the brain stem and spinal cord suggests pre- and postsynaptic localization in both glutamatergic and glycinergic synapses (Brooke et al., 2004a). There is also evidence for Kv3.4 expression in muscle and pancreas (Abbott et al., 2001; Gopel et al., 2000), albeit the composition of Kv3 channels that include Kv3.4 subunits in these tissues is unclear. In contrast, Kv3.4 is the dominant Kv3 subunit in rat dorsal root ganglion (DRG) neurons, where it has been detected in axons, somata and synaptic terminals (Alexander et al., 2022; Chien et al., 2007; Muqeem et al., 2018; Ritter et al. 2012, 2015a; Zemel et al., 2018). Results in mice show a similar prominence of Kv3.4 in somatosensory neurons. Deep sequencing and electrophysiological analyses of these neurons showed that Kv3.4 is preferentially expressed in both peptidergic and non-peptidergic neurons, which express Kv3.1, Kv3.2 and Kv3.3 at significantly lower levels (Zheng et al., 2019). Therefore, Kv3.4 may exist as a homotetrameric assembly in these DRG neurons. This work also showed that, among eight distinct DRG neuron subtypes, Aδ low-threshold mechanoreceptors exhibit the highest expression of Kv3.4 and significantly lower expression of other Kv3 isoforms (Zheng et al., 2019). Strongly supporting a homotetrameric assembly of Kv3.4 channels in DRG neurons, the native high voltage-activating A-type current in these neurons and heterologously expressed Kv3.4 currents exhibit similar biophysical properties (Alexander et al., 2022; Fineberg et al., 2012; Ritter et al., 2012).

Although all Kv3 channel subtypes generate high-voltage activating currents, they exhibit significant differences with respect to inactivation kinetics and the underlying mechanisms of inactivation (Kaczmarek and Zhang, 2017; Rudy and McBain, 2001). While Kv3.1 and Kv3.2 undergo little to no inactivation within a physiological time range of tens of milliseconds, Kv3.3 and Kv3.4 undergo substantial inactivation. Dephosphorylated Kv3.4 channels generate outward currents with the fastest trajectory of inactivation, which is profound in about 100 ms at room temperature and depolarized membrane potentials (both heterologously and natively expressed) (Alexander et al., 2022; Fineberg et al., 2012; Ritter et al., 2012). Also, it has been unambiguously established that the Kv3.4 channel undergoes strict open-state inactivation (OSI), possibly mediated by a ball-and-chain N-type mechanism in which the channel’s N-terminal inactivation domain (NTID) bearing a net positive charge plugs the inner mouth of the open K^+^-selective pore (Fineberg et al., 2012; Hoshi et al., 1990). Kv3.4 OSI, however, is not a static property. The first 28 amino acids of the Kv3.4 protein constitute the NTID, which includes cysteine (C6 and C24) and serine (S8, S9, S15 and S21) residues that are established targets of post-translational modifications. Thus, Kv3.4 OSI is dramatically slowed by cysteine modifications involving sulfhydration, oxidation, and hemin coordination, and serine modifications upon multisite phosphorylation by PKC (Antz et al., 1999; Beck et al., 1998; Coburger et al., 2020; Covarrubias et al., 1994; Ritter et al., 2012; Ruppersberg et al., 1991; Yang et al., 2019). Kv3.3 N-type inactivation is also modulated by PKC (Desai et al., 2008), and other Kv channels exhibit modulation of N-type inactivation by other kinases and regulatory subunits (Drain et al., 1994; Roeper et al. 1997, 1998). Here, we focus on Kv3.4 NTID phosphorylation by PKC, with emphasis on the structural, biophysical and physiological impacts of this modification. Upon phosphorylation, the presumed NTID binding at the channel’s internal mouth is harder and unbinding is easier (i.e., OSI is destabilized), resulting in delayed onset of inactivation and accelerated recovery from inactivation (Beck et al., 1998).

**Fig. 4.**
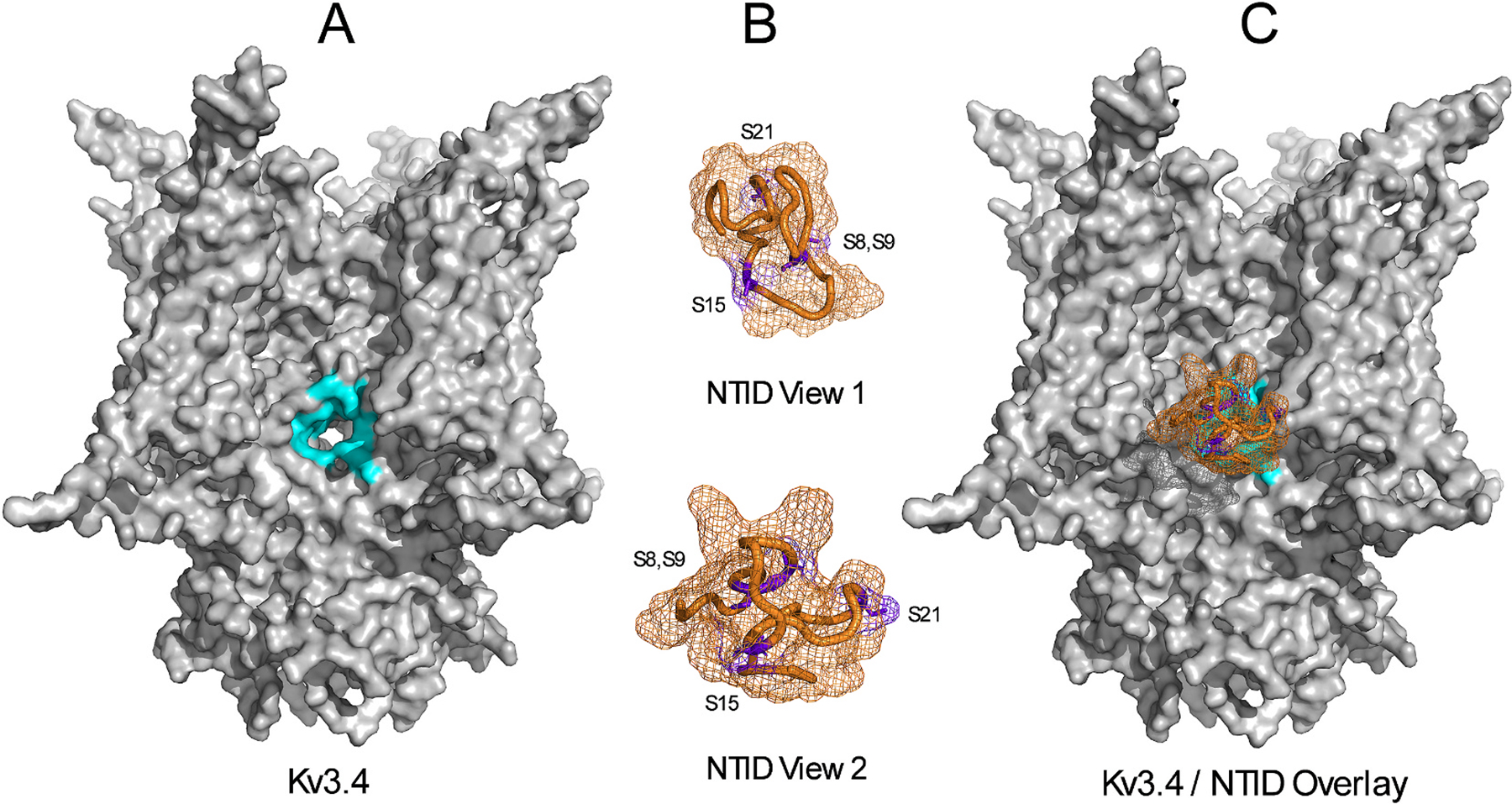
The Kv3.4 NTID faces a steric clash at the lateral intracellular windows of the Kv3.4 channel. **A)** Kv3.4 model highlighting the lateral opening (cyan) that the NTID must cross to reach the intracellular mouth of the pore. This model does not include the free NTIDs. **B)** The NMR structure of the Kv3.4 NTID displayed in two orientations (PDB ID: 1ZTN). For improved visualization of phosphorylation sites (highlighted in purple), these structures are displayed enlarged relative to the image of the Kv3.4 model on panel A. **C)** Overlay of the Kv3.4 model plus the appropriately scaled View 2 of the NTID. This image shows that the lateral opening is not large enough to allow the NTID to enter the internal vestibule of the Kv3.4 channel and reach its internal mouth. The Kv3.4 model was created using AlphaFold3 (Jumper et al., 2021) and the visualizations were created using PyMOL (The PyMOL Molecular Graphics System, Version 3.0 Schrödinger, LLC).

NMR studies of the untethered Kv3.4 NTID peptide revealed a compact clover-like structure in its dephosphorylated state, which upon phosphorylation at S8 and S21 became mostly disordered. Therefore, phosphorylation does not simply neutralize the net positive charge of the NTID to disrupt OSI. Instead, another study demonstrated that the contributions of the Kv3.4 NTID phosphorylation sites to OSI disruption are not equivalent (Beck et al., 1998). S8 and S9 play more significant roles than S15 and S21, and these contributions are not additive. More likely, cooperative interactions drive a phosphorylation-induced unfolding of the NTID that is ultimately responsible for a dramatic destabilization of OSI. However, consideration should be given to caveats of this model. The NTIDs of the Kv1 and Kvβ1 subunits exhibit disordered structures, yet they are fast and effective inactivators (Antz et al., 1997; Wissmann et al., 1999). In contrast, the NTID of Kv3.4 appears to be the most effective when it adopts a compact clover-like structure in its dephosphorylated state, becoming ineffective when it gains negative charges and adopts a disordered conformation upon phosphorylation. This is intriguing because structural modeling based on the available NMR and cryo-EM structures demonstrate that the Kv3.4 NTID in its compact structure would not fit through the lateral cytoplasmic windows of the channel in its open conformation to reach the inner mouth of the pore (Fig. 4). Nevertheless, the data suggest that the dephosphorylated Kv3.4 NTID binds quickly and tightly to an open pore site to induce stable OSI (Beck et al., 1998). Accordingly, the synthetic Kv3.4 NTID peptide retains a robust ability to induce fast inactivation, indicating that tethering to the rest of the channel protein is not necessary for structure and function (Antz et al., 1999; Murrell--Lagnado and Aldrich, 1993). Two hypotheses may help resolve these apparent inconsistencies. First, the lateral windows may widen substantially as the pore opens allowing access of the compact dephosphorylated Kv3.4 NTID to the channel’s inner mouth. Second, the dephosphorylated Kv3.4 NTID may plug the lateral windows rather than the pore’s inner mouth. The lateral windows may change shape when the channel opens, thereby exposing a binding site for the NTID. Since the lateral windows may contribute to a conformational change of the cytoplasmic T1 domain that is -as discussed previously- coupled to activation gating, these hypotheses are feasible and should be evaluated to finally determine how the Kv3.4 channel inactivates, and how the inactivation process is modulated by cytosolic signaling.

Given their inactivation properties and modulation by cell signaling, Kv3.4 subunits may finetune the voltage- and time-dependent properties of the heteromeric Kv3 channels that govern fast spiking and AP repolarization rate in diverse neuronal subtypes (Alexander et al., 2022; Baranauskas et al., 2003; Kaczmarek and Zhang, 2017; Rowan and Christie, 2017). Modulation of AP repolarization rate would be especially important at synaptic terminals because it can dictate the magnitude of evoked Ca^++^– dependent synaptic transmission (Geiger and Jonas, 2000; Rowan and Christie, 2017). The physiological roles of the native Kv3.4 channel have been best characterized in rodent DRG neurons because they exhibit dominant expression of Kv3.4 subunits (relative to other Kv3 subunits) and high voltage K^+^ currents with properties characteristic of Kv3.4 homomeric assemblies (Alexander et al., 2022; Ritter et al., 2012; Zheng et al., 2019). An early study demonstrated that Kv3.4 channels in small diameter DRG neurons from rats undergoes a loss of fast inactivation upon activation of PKC by either phorbol esters or by G-protein coupled receptor (GPCR) ligands present in an inflammatory cocktail including histamine, serotonin and bradykinin (Ritter et al., 2012). The latter experiments also showed that inactivation modulation of Kv3.4 occurs in membrane-delimited manner, which is consistent with the presence of a multiprotein signaling complex including a GPCR, PKC, and Kv3.4. A similar loss of Kv3.4 fast inactivation was also observed upon inhibition of calcineurin, suggesting that this Ca^++^-dependent phosphatase works in concert with the basal activity of PKC to modulate Kv3.4 gating in DRG neurons (Zemel et al., 2017).

Further work showed that Kv3.4 knockdown in DRG neurons broadens the AP by slowing the repolarization phase, and that the treatments that activate PKC also shorten the AP by accelerating the repolarization phase (Alexander et al., 2022; Ritter et al., 2012). This is compelling evidence for a major role of Kv3.4 as a driver of AP repolarization. Consistent with the expected impact of AP duration and the rate of repolarization on evoked synaptic transmission, subsequent work showed that inhibition of presynaptic Kv3.4 with low concentrations of TEA and 4-AP in an intact spinal cord preparation potentiates glutamatergic synaptic transmission in the superficial dorsal horn (Muqeem et al., 2018). Therefore, the expression and modulation of Kv3.4 gating tune the rate of AP repolarization to modulate synaptic transmission. Independently, another study reached this conclusion by showing that the availability of a fast inactivating Kv3 channel drives flexible synaptic signaling in cerebellar stellate interneurons (Rowan and Christie, 2017).

A subsequent study expressed recombinant Kv3.4 variants in cultured DRG neurons to directly show that AP width is (as previously suspected) linked to Kv3.4 NTID phosphorylation (Alexander et al., 2022). The constitutively phosphorylated Kv3.4 variant (phosphomimic with all NTID PKC sites mutated to aspartate) accelerated AP repolarization (consequently shortening the AP). In contrast, the constitutively dephosphorylated Kv3.4 (phosphonull with all NTID PKC sites mutated to alanine) slowed AP repolarization (consequently broadening the AP). In agreement with the link between NTID phosphorylation and the rate of AP repolarization, overexpression of Kv3.4 wild type (i.e., phosphorylatable because all NTID PKC sites are available) in DRG neurons yielded APs with intermediate widths, between those observed with the Kv3.4 phosphomimic and Kv3.4 phosphonull. Establishing this link, however, did not explain how the loss of Kv3.4 fast inactivation affects AP repolarization rate and width, and under what condition would this modulation play a dynamic role on the width of the AP. A slower development of Kv3.4 inactivation over a time scale of tens to hundreds of milliseconds is not likely to play a major role on the repolarization of the AP, which takes place over a time scale of a few milliseconds. The peak Kv3.4 conductance, which is proportional to the expression and functional availability of Kv3.4 channels, is more likely to determine AP repolarization rate and width. Accordingly, Kv3.4 NTID phosphorylation increases the Kv3.4 peak conductance by preventing transitions to the inactivated state and, thereby, shortens the AP. This relation may, however, change if the functional availability of Kv3.4 channels is dynamically modulated.

The AP is generally regarded as a binary all-or-none and relatively rigid response that mainly encodes information in the timing, frequency and patterns of AP firing. However, AP waveforms are plastic. A wealth of studies has shown that the APs from diverse neural preparations undergo broadening in a use- and frequency-dependent manner (Aldrich et al., 1979; Gainer et al., 1986; Geiger and Jonas, 2000; Jackson et al., 1991; Liu et al., 2017; Ma & Koester, 1995, 1996). Therefore, APs may also dynamically encode analog information in their shape and duration to dial the magnitude of evoked neurotransmitter release at synapses. In support of this idea, use-dependent spike broadening (UDSB) is a determining factor of activity-dependent synaptic facilitation (Geiger and Jonas, 2000; Regehr, 2012). APs from DRG neurons undergo substantial UDSB, which increases with an increase in the frequency of stimulation ranging between 1 and 20 Hz (Alexander et al., 2025; Liu et al., 2017). To understand the physiological role of Kv3.4 and the biophysical underpinnings of this role, a recent report showed that the sensitivity of the AP to undergo UDSB depends on phosphorylation of the Kv3.4 NTID in DRG neurons (Alexander et al., 2025). Expression of the Kv3.4 phosphomimic variant sharpens the AP and protects against UDSB. By contrast, expression of the Kv3.4 phosphonull variant greatly increases the sensitivity of the AP to UDSB, albeit the initial AP width is like that of APs from DRG neurons expressing the phosphomimic Kv3.4 variant. Expression of the Kv3.4 wild type (with all PKC sites available), on the other hand, conferred intermediate sensitivity to UDSB because, as mentioned above, it can exist as a heterogeneous population of Kv3.4 channels composed of both phosphorylated and dephosphorylated subunits. To explain this pattern, further experiments revealed that whereas the Kv3.4 phosphonull variant exhibits slow recovery from inactivation and correspondingly cumulative inactivation under the repetitive stimulation conditions that induce UDSB, the Kv3.4 phosphomimic variant exhibits fast recovery from inactivation and no cumulative inactivation under identical stimulation conditions. Again, the Kv3.4 wild type displayed intermediate inactivation properties. Considering these changes, dynamic clamp experiments with individual DRG neurons demonstrated real time switching from UDSB-sensitive APs, following virtual expression of Kv3.4 phosphonull, to UDSB-resistant APs, following virtual expression of Kv3.4 phosphomimic (Alexander et al., 2025). Demonstrating that UDSB depends on the expression of Kv3.4 in DRG neurons, in vitro knock out of Kv3.4 yields prolonged APs (as observed previously) that are also UDSB-resistant. Moreover, the prolonged width of these APs marks a ceiling corresponding to the maximal UDSB conferred by the expression of Kv3.4 phosphonull under dynamic clamp conditions (Alexander et al., 2025). The results of this work solidify three significant conclusions about the function and modulation of the Kv3.4 channel. First, Kv3.4 is a major driver of fast AP repolarization in DRG neurons. Second, phosphorylation of the Kv3.4 NTID dictates the rate of recovery from inactivation and the sensitivity of Kv3.4 channels to undergo cumulative inactivation under conditions of repetitive firing at frequencies ≥1 Hz. Third, the phosphorylation-dependent sensitivity of Kv3.4 to use-dependent cumulative inactivation dictates the sensitivity of APs to undergo UDSB. It is then reasonable to hypothesize that this mechanism would dynamically modulate short-term activity-dependent synaptic plasticity.

**Fig. 5.**
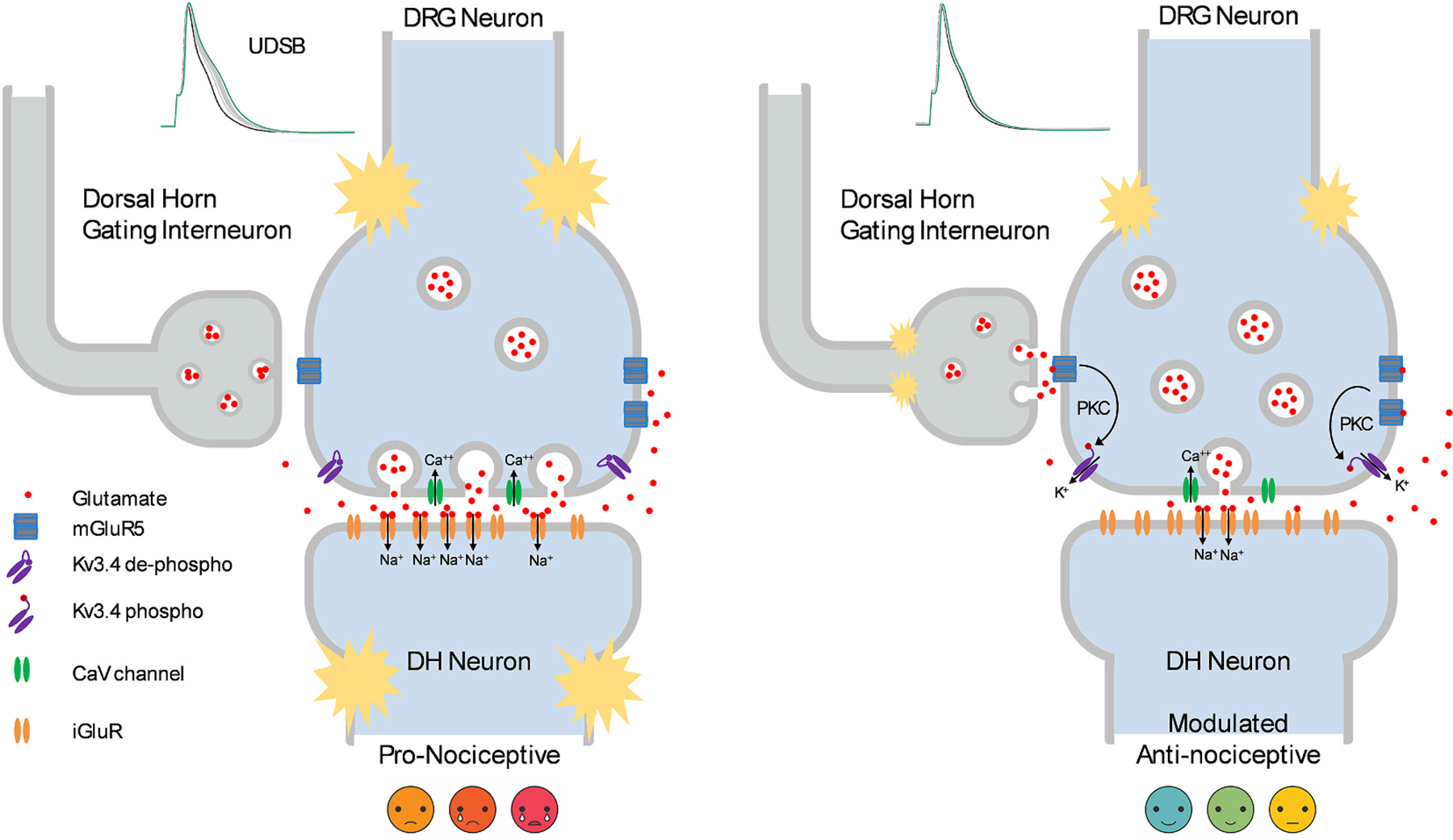
Working model of synaptic plasticity governed by phosphorylation of the Kv3.4 channel in dorsal horn synapses of the spinal cord. A nociceptive stimulus may cause repetitive DRG neuron spiking at frequencies that induce cumulative Kv3.4 inactivation and UDSB (left). Consequently, the dorsal horn (DH) glutamatergic synapse that receives this input in laminae I and II undergoes activity-dependent facilitation to relay a strong pro-nociceptive signal. To regulate this signal, released glutamate may also act on presynaptic mGluR5 to trigger a Gq/11-dependent signaling cascade that activates PKC, which would then phosphorylate the Kv3.4 NTID. This phosphorylation destabilizes Kv3.4 inactivation, shortens the AP and opposes UDSB. As a result, the modulated synapse is now depressed and nociceptive signaling is dampened. Glutamatergic gating interneurons in the DH could similarly activate presynaptic mGluR5 to modulate nociceptive signaling.

If PKC is a central player in the mechanism that modulates the plasticity of UDSB and its impact on synaptic transmission, what GPCR and which pathways would initiate the signaling cascade? Since Kv3.4 channels modulate glutamatergic synapses in the spinal cord’s superficial dorsal horn, a plausible scenario involves two possible pathways (Fig. 5). Nociceptive stimulation evokes glutamate release mainly from C and Aδ fibers, which quickly acts on postsynaptic glutamate receptors at the postsynaptic membrane of the secondary sensory neuron to relay the nociceptive signal. Free glutamate outside the synaptic cleft, however, would be either taken up by astrocytes or bind to presynaptic metabotropic glutamate receptors (e.g., Gq/11-coupled mGluR5) creating a negative feedback loop that would oppose the pro-nociceptive short-term synaptic facilitation resulting from UDSB (Fig. 5). That is, activation of mGluR5 in a multiprotein signaling complex would trigger PKC activation to phosphorylate the NTID of the presynaptic Kv3.4 and, consequently, switch the AP response from UDSB-sensitive to UDSB-resistant. This would have an anti-nociceptive effect by preventing overactivation and possible long-term postsynaptic potentiation of the nociceptive glutamatergic synapse. Axo-axonic synapses from glutamatergic gating interneurons in the spinal cord may similarly act on the nociceptive excitatory synapse to favor the UDSB-resistant response and the resulting anti-nociceptive effect (Fig. 5). These pathways constitute an attractive working model of pain modulation for several reasons. First, it is economical because glutamate plays a dual role, simultaneously transmitting and regulating nociceptive signaling. Second, the model is supported by studies that have demonstrated the role of group I mGluRs in peripheral and central pain modulation, and the expression of mGluR5 in rodent DRG neurons (Bhave et al., 2001; Montana and Gereau, 2011; Xie et al. 2017, 2023). Third, it is consistent with models of presynaptic modulation in other central synapses (Chu et al., 2014; Nicoll and Schmitz, 2005; Niswender and Conn, 2010). Finally, the model is also consistent with a gated mechanism of pain modulation involving glutamatergic dorsal horn interneurons potentially acting on synapses from Aδ LTMRs (Koch et al., 2018). More complex and non-mutually exclusive mechanisms may rely on descending modulation by volume transmission involving monoamines that activate canonical Ca^++^-dependent PKC signaling and thereby modulate downstream targets, such as Kv3.4 (Bannister and Dickenson, 2016). Also, other factors and alternate signaling mechanisms may modulate Kv3.4 N-type inactivation and expression and consequently determine UDSB and synaptic facilitation; for instance, hemin, cysteine oxidation, sulfhydration and PKC-ε (Bannister and Dickenson, 2016; Coburger et al., 2020; Yang et al., 2019; Zemel et al., 2021).

Beyond function, this working hypothesis provides a framework to explain pathological states that have implicated Kv3.4 dysfunction. In a rodent nerve ligation model of neuropathic pain, an early study found downregulated expression of Kv3.4 in the DRG (Chien et al., 2007). This study also found that knocking down peripheral Kv3.4 expression with an antisense oligonucleotide selectively induces mechanical allodynia in naïve rats (Chien et al., 2007). This result is consistent with the dominant expression of Kv3.4 in Aδ-LTMR fibers (Zheng et al., 2019). In another model of neuropathic pain induced by cervical spinal cord injury, DRG neurons become hyperexcitable, the surface expression of Kv3.4 is inhibited, and the APs of DRG neurons lose their Kv3.4-dependent sensitivity to width modulation upon PKC activation (Ritter et al., 2015a). Although the mechanism responsible for Kv3.4 inhibition is still unknown, it involves an injury induced inhibition of calcineurin, which may cause hyperphosphorylation of Kv3.4 (Zemel et al., 2017). Taken together, these observations suggest a significant contribution of peripheral Kv3.4 dysregulation to a complex mechanism involving peripheral and central maladaptive changes that are ultimately responsible for the neuropathic pain induced by traumatic injuries (Brown et al., 2022; Gold and Gebhart, 2010; Walters, 2012).

Dysregulation of Kv3.4 has also been implicated in other pathologies. Through an unknown mechanism, there is evidence to suggest that upregulated expression of Kv3.4 in the brain may contribute to synaptic dysfunction at an early stage of Alzheimer’s disease (Boscia et al., 2017; Choi and Abbott, 2007; Yeap et al., 2022). Also, a pathogenic variant of the promiscuous accessory protein MiRP2 causes Kv3.4 dysfunction in human skeletal muscle, which is responsible for the periodic paralysis experienced by patients who carry this variant (Abbott et al., 2001). Lastly, many human *KCNC4* variants have been recently reported on the ClinVar database (https://www.ncbi.nlm.nih.gov/clinvar/); however, most of them have uncertain significance, and many are likely benign. Therefore, the clinical significance of KCNC4 variants is yet to be established. This contrasts with a growing number of pathogenic *KCNC1*, *KCNC2* and *KCNC3* variants causing a wide range of developmental and neuropsychiatric anomalies.

## 6. Concluding remarks and perspective

Over the past fifteen years, we have devoted significant efforts toward elucidating the function, structure and modulation of Kv3 channels. This work was inspired and progressively stimulated by three major developments in this field. First, the identification of a growing number of neuropsychiatric disorders caused by GoF and LoF variants of *KCNC1*, *KCNC2* and *KCNC3*. Second, an increased interest in the discovery of specific small molecule modulators to treat these disorders. Third, foundational work that suggests a major role for homomeric Kv3.4 channels in pain signaling. From the progress made, we have concluded that: 1) the cytoplasmic T1 domain is a major co-determinant of fast voltage-dependent gating and that pathogenic Kv3.2 variants with discrete mutations in this region disrupt this role; 2) the mechanism of action of imidazolidinedione derivatives (AUT1 and AUT5) involves occupancy of a binding pocket with unique features and a surprising conformational change of the extracellular “turret” region, which explains the highly specific positive modulation of Kv3.1 and Kv3.2 channels by AUT1 and AUT5; 3) Kv3.4 channels expressed in nociceptors and mechanoreceptors drive the repolarization of the AP and dynamically modulate UDSB in a manner that depends on phosphorylation of the Kv3.4 NTID; 4) phosphorylation of the Kv3.4 NTID speeds up recovery from Kv3.4 inactivation to oppose UDSB; and 5) UDSB modulation by Kv3.4 may modulate somatosensory signaling in the spinal cord through presynaptic mGluR5 pathways involving retrograde signaling, gating interneurons and descending projections.

To advance the field further, important questions must be answered at the structural, molecular, cellular and organismal levels. For instance, what dynamic gating rearrangements does the Kv3 T1 domain undergo to regulate fast voltage-dependent gating? What dynamic interactions between the VSD and the PD underlie the positive modulation of Kv3.1 and Kv3.2 by imidazolidinedione derivatives? How would a better understanding of these compounds’ transduction mechanism inform the development of new derivatives with improved efficacy? Does the Kv3.4 NTID bind to the lateral cytoplasmic windows or the internal mouth of the channel to cause fast inactivation? How do heterogeneous nociceptors and mechanoreceptors utilize specific Gq/11 coupled receptors to modulate Kv3.4 inactivation and UDSB, which would modulate activity-dependent synaptic transmission involved in somatosensory transduction? Does phosphorylation of the Kv3.4 NTID and its modulation by other cellular factors and pathways impact somatosensensory signaling in vivo? Elucidating these questions would pave the way toward translating this knowledge into innovative therapeutic approaches to treat Kv3 encephalopathies, psychiatric maladies and neuropathic pain.

## Data Availability

No data was used for the research described in the article.
